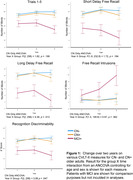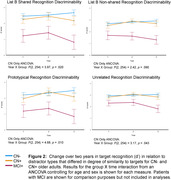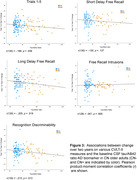# The Nature of Episodic Memory Decline in Preclinical Alzheimer’s Disease

**DOI:** 10.1002/alz.093414

**Published:** 2025-01-03

**Authors:** Anjali Sharma, Melanie L Johnson, Kelsey R. Thomas, Douglas R. Galasko, Mark W. Bondi, Tamar H. Gollan, Guerry M. Peavy, Diane M. Jacobs, David P. Salmon, Dean C. Delis

**Affiliations:** ^1^ University of California, San Diego, San Diego, CA USA; ^2^ Shiley‐Marcos Alzheimer’s Disease Research Center, San Diego, CA USA; ^3^ San Diego State University, San Diego, CA USA; ^4^ VA San Diego Healthcare System, San Diego, CA USA; ^5^ University of California, San Diego, La Jolla, CA USA; ^6^ Shiley‐Marcos Alzheimer’s Disease Research Center, La Jolla, CA USA; ^7^ Department of Neurosciences, University of California San Diego, La Jolla, CA USA; ^8^ University of California San Diego, Department of Neurosciences, La Jolla, CA USA; ^9^ Department of Psychiatry, University of California, San Diego, San Diego, CA USA

## Abstract

**Background:**

Alzheimer’s disease (AD) pathology (e.g., beta‐amyloid plaques, tau neurofibrillary tangles) accumulates before the emergence of cognitive deficits that lead to a diagnosis of mild cognitive impairment (MCI) or dementia. Early vulnerability of medial temporal lobe structures to AD suggests that subtle episodic memory decline should be among the first cognitive markers of the disease. Therefore, we examined the relationship between 2‐year change in episodic memory and baseline cerebrospinal fluid (CSF) biomarkers of AD in cognitively normal (CN) older adults.

**Methods:**

Episodic memory was assessed with the California Verbal Learning Test, Second Edition (CVLT‐II), a sensitive 16‐item list learning test that measures multiple aspects of memory proficiency. We administered the CVLT‐II at baseline, 1‐year, and 2‐year evaluations to older adults who were CN and CSF AD biomarker‐negative (CN–; *n* = 94) or positive (CN+ or preclinical AD; *n* = 38; CSF tau/Aβ42 ratio>.609) at baseline. We compared groups on change over time on total recall on List A learning trials 1‐5, list B free recall, short‐ and long‐delay free and cued recall, intrusion errors, and recognition discriminability. We also examined target recognition (d’) in relation to distractor types that differed in degree of similarity to targets.

**Results:**

Group X time repeated measures ANCOVAs controlling for age and sex showed significantly greater 2‐year decline in CN+ than CN‐ on CVLT‐II measures of long‐delay free recall (interaction: F(2,256) = 4.39;p<.05) and recognition discriminability overall (interaction: F(2,256) = 3.08;p<.05) (Fig. 1) and against most distractor types (p’s<.05) (Fig. 2). Higher tau/Aβ42 ratio (i.e., more AD‐like) in CN older adults was associated with greater decline on long‐delay free recall (*r*(130) = –.205;*p*<.02) and recognition discriminability (*r*(130) = –.215;*p*<.02) (Fig. 3). However, magnitude of decline only marginally categorized individuals as CN‐ vs. CN+ in logistic regression analyses (95% specificity, but only 21% sensitivity).

**Conclusion:**

Subtle decline in episodic memory is evident during the preclinical phase of AD, affecting both delayed recall and recognition consistent with medial temporal lobe dysfunction. In addition, greater preclinical memory decline is associated with higher levels of an AD biomarker in CSF. Further research will determine if these memory changes can serve as a cognitive marker of preclinical AD.